# Tuberculosis Transmission Attributable to Close Contacts and HIV Status, Malawi

**DOI:** 10.3201/eid1205.050789

**Published:** 2006-05

**Authors:** Amelia C. Crampin, Judith R. Glynn, Hamidou Traore, Malcolm D. Yates, Lorren Mwaungulu, Michael Mwenebabu, Steven D. Chaguluka, Sian Floyd, Francis Drobniewski, Paul E.M. Fine

**Affiliations:** * London School of Hygiene and Tropical Medicine, London, United Kingdom;; † Karonga Prevention Study, Chilumba, Malawi;; ‡ Health Protection Agency National Mycobacterium Reference Laboratory, London, United Kingdom

**Keywords:** tuberculosis, HIV, RFLP, tuberculosis transmission, Africa, research

## Abstract

We conducted the first molecular study of tuberculosis (TB) to estimate the role of household contact and transmission from HIV-positive putative source contacts (PSCs) in a high HIV-prevalence area. TB patients in a long-term population-based study in Malawi were asked about past contact with TB. DNA fingerprinting was used to define clusters of cases with identical strains. Among 143 epidemiologically defined PSC-case pairs, fingerprinting confirmed transmission for 44% of household and family contacts and 18% of other contacts. Transmission was less likely to be confirmed if the PSC were HIV positive than if he or she was HIV negative (odds ratio 0.32, 95% confidence interval [CI] 0.14–0.74). Overall, epidemiologic links were found for 11% of 754 fingerprint-clustered cases. We estimate that 9%–13% of TB cases were attributable to recent transmission from identifiable close contacts and that nearly half of the TB cases arising from recent infection had acquired the infection from HIV-positive patients.

## Abstract 2

In this population, ≈90% of *M. tuberculosis* infections were transmitted by casual contact, and nearly half from HIV-positive patients.

The HIV epidemic has dramatically increased tuberculosis (TB) incidence. The magnitude of this effect depends in part on the relative infectiousness of HIV-infected TB patients: they are less likely to have smear-positive disease and may be infectious for a shorter period than other patients since they have higher death rates and may seek health care earlier (*1*). Several studies have found that household contacts of HIV-positive patients had lower rates of *Mycobacterium tuberculosis* infection than those of HIV-negative patients, even after adjusting for sputum smear status of the cases and HIV status of the contacts (*2**–**4*), though other studies have found no differences in infection rates (*5**–**7*).

DNA fingerprinting can be used to identify clusters of TB patients that share *M. tuberculosis* strains with identical patterns and to estimate when transmission occurred. To date, DNA fingerprinting studies comparing transmission from HIV-positive and HIV-negative patients have been small, and the differences have not been significant (*8**,**9*).

Studies can investigate sources of *M. tuberculosis* infection by seeking epidemiologic links within fingerprint-defined clusters or by comparing the DNA fingerprints of epidemiologically linked persons (*8**–**10*). In this study, we combine these 2 approaches to analyze the only long-term population-based molecular epidemiologic study of TB in an area with a high prevalence of HIV. Novel methods were used to estimate the proportion of TB in the population that is attributable to transmission from known contacts and from HIV-positive patients.

## Methods

Since 1986, as part of the Karonga Prevention Study in northern Malawi, patients in whom TB was suspected have been identified by using enhanced passive surveillance. Project staff are based at peripheral clinics and the district hospital to examine anyone with chronic cough or enlarged lymph nodes. Patients in whom TB is suspected are also identified in the course of other studies, including household visits to TB patients, although in practice most patients come to the clinic or hospital. Sputum is taken for smear microscopic examination and culture, and material from lymph node biopsy specimens, ascites, and pleural fluid is also cultured when available (*11*). DNA fingerprinting has been carried out on cultures from all TB patients since late 1995 (*12*). Cultures that macroscopically resemble *M. tuberculosis* are sent to the Health Protection Agency Mycobacterium Reference Laboratory, London, United Kingdom, for species identification and drug sensitivity testing. *M. tuberculosis* specimens are fingerprinted by using IS*6110* restriction fragment length polymorphism (RFLP), following standard procedures (*13*). Spoligotyping (*14*) is performed on strains with <5 bands on the RFLP pattern. Treatment follows Malawi National TB Control Programme guidelines. TB patients were tested for HIV after counseling and if consent was given. No antiretroviral treatment was available at the time of the study (*11*).

Since 1997, at the time of diagnosis, all TB patients have been asked about persons they knew who had had TB, either in their family or household (at any time) or among other acquaintances (in the last 5 years) (*15*). Details gathered on these putative source contacts (PSCs) allowed them to be identified within the project database: ≈90% of named PSCs who were said to have had TB in the district within the previous 5 years were confirmed as having been treated for TB. In addition, all persons seen in the district during long-standing epidemiologic studies are asked about their current and past residences and their parents, allowing genetic linkages and household histories to be constructed. PSCs identified only from the epidemiologic database were included in this study if they were first-degree relatives or half siblings of the patient or if they were documented as having lived in the same household as the patient at the time that the PSC had TB.

DNA fingerprints of cultures from all case-PSC pairs were compared by computer (Gelcompar 4.1, Applied Maths, Kortrijk, Belgium) and checked visually (*12*). Transmission was "confirmed" if the pair had identical strains, or if the RFLP patterns differed by 1 to 4 bands and the later strain was the first or only example of the new pattern in the dataset (*12**,**16*). Since >1 PSC was identified for some patients, the analysis was repeated excluding PSCs with smear-negative results or extrapulmonary disease, and, if >1 PSC was smear-positive, choosing the most likely source of the infection by selecting the most closely matched strains and the closest contacts (e.g., contacts within the household were considered closer than nonhousehold contacts). We have previously explored RFLP pattern evolution among the first 80 such pairs with smear-positive PSCs (to 2001) (*12*). Here, in a larger dataset, we explored risk factors associated with a named PSC being the confirmed source of transmission and estimate the proportion of TB in the population attributable to contact with a smear-positive household or other close family member and the relative contribution of HIV-positive and HIV-negative patients to transmission.

In addition, we estimated the proportion of RFLP-defined links that can be explained epidemiologically. Strains were defined as clustered if the RFLP pattern was shared by >2 patients. The proportion of patients in RFLP-defined clusters for whom epidemiologic links were known was calculated, and any variation with cluster size or band number was investigated. Assuming 1 index case per cluster, we calculated the proportion of secondary cases within clusters for which an epidemiologically linked source could be identified (*17*).

## Statistical Analysis

Comparison of proportions used χ^2^ tests, or exact tests when numbers were small. Odds ratios were calculated by using logistic regression. To calculate the proportion of cases in the population attributable to different types of contact (the population attributable fraction [PAF]), we adapted the formula PAF = p´(RR-1)/RR where p´ is the prevalence of the exposure (history of contact) in the cases, and RR is the relative risk of TB in those who are exposed compared to those who are not exposed (*18*). The expression (RR-1)/RR is algebraically equivalent to the risk difference percent, (*r*_1_–*r*_0_)/*r*_1_, where *r*_1_ is the risk in the exposed, and *r*_0_ is the risk in the unexposed. We do not know RR, *r*_1_, or *r*_0_, but the risk difference percent is equivalent to the proportion of epidemiologically linked cases for which transmission from a PSC is confirmed (since this represents the proportion of cases in the exposed group that were caused by the exposure). PAF is thus calculated as the proportion of case-PSC pairs for which transmission was confirmed, multiplied by the prevalence of exposure (having a PSC) among the cases. To estimate the contribution of HIV-positive patients to onward transmission, we adjusted the relative probabilities of transmission being confirmed from HIV-positive and HIV-negative PSCs, by the proportion of smear-positive TB patients in the population who were HIV positive.

### Ethics permission

Permission for the study was received from the Malawi National Health Sciences Research Committee and the ethics committee of the London School of Hygiene and Tropical Medicine.

## Results

From late 1995 to early 2003, a total of 1,248 culture-positive TB patients were identified in Karonga District. Successful RFLP fingerprints were available on 1,194 isolates from 1,044 patients. After excluding 25 fingerprints because laboratory error was suspected (*12*), 1,029 patients had RFLP results: 74% were clustered (*19*). The isolates from 81 persons had <5 bands, and spoligotypes were available on 64 of these. HIV results were available for 61%, of whom 65% were positive.

### Transmission Confirmation in Epidemiologically Defined Case-PSC Pairs

Fingerprints were available for 200 case-PSC pairs, of whom 51 had identical strains and 8 more had similar strains that were likely to be attributable to transmission between the 2 persons (Table 1). Transmission was no more likely to be confirmed if the information came from the patient's history only or from the epidemiologic database only, but was more likely if the information came from both sources (p = 0.05). Transmission was more likely if PSCs had smear-positive TB than if they had smear-negative or extrapulmonary TB (p = 0.06). Of the 7 pairs with confirmed matches and RFLP patterns with <5 bands, spoligotypes for both members of the pair were available for 3; they were identical for 2, and different for the third (a strain with 4 bands). The pair with different spoligotypes and a pair with similar but not identical RFLP patterns, with 1 band for the PSC and 4 bands for the patient (and missing spoligotypes), were excluded from further analyses.

**Table 1:** Comparison of *Mycobacterium tuberculosis* strains of index cases and putative source contacts (PSCs)

		Comparison of strains, no. (%)				
		Identical	1–4 bands different and first example of new strain	1–4 bands different and not first example	>4 bands different	Total
Total		51 (25.5)	8 (4.0)	15 (7.5)	126 (63.0)	200
Source of linking information						
	Database only	9 (25.7)	1 (2.9)	5 (14.3)	20 (57.1)	35
	History only	14 (16.5)	4 (4.7)	6 (7.1)	61 (71.8)	85
	Both	28 (35.0)	3 (3.8)	4 (5.0)	45 (56.3)	80
Characteristic of PSC in case-PSC pairs						
	Smear-positive pulmonary	48 (28.7)	7 (4.2)	15 (9.0)	97 (58.1)	167
	Smear-negative pulmonary	2 (9.1)	1 (4.6)	0 (0.0)	19 (86.4)	22
	Extrapulmonary	1 (9.1)	0 (0.0)	0 (0.0)	10 (90.9)	11
No. bands in RFLP* of PSC						
	<5	6 (40.0)	1 (6.7)	0 (0.0)	8 (53.3)	15
	5–10	17 (22.7)	6 (8.0)	8 (10.7)	44 (58.7)	75
	>10	28 (25.5)	1 (0.9)	7 (6.4)	74 (67.3)	110

*RFLP, restriction fragment length polymorphism.

When only smear-positive PSCs were used and the most likely source of transmission was selected, RFLP confirmation of transmission was much more likely for household and family PSCs (44%) than for other PSCs (friends, neighbors or colleagues, 18%, Table 2). Transmission was confirmed for 8 (62%) of 13 spouse pairs, and for 12 (48%) of 25 persons who nursed the sick patient or shared a sleeping dwelling with them. Transmission was less likely to be confirmed from male than from female PSCs, and less likely from HIV-positive PSCs than from HIV-negative PSCs (Table 2). The effect of sex of the PSC (odds ratio [OR] 0.39, 95% confidence interval [CI] 0.19–0.81) was reduced by adjusting for closeness of contact (OR 0.46, 95% CI 0.21–0.99) and was no longer significant after adjusting for HIV status of the PSC (OR 0.56, 95% CI 0.25–1.2). The effects of closeness and of HIV status of the PSC became slightly stronger when each factor was adjusted for: adjusted OR 4.6 (1.7–12.3) for family contacts and 4.1 (1.6–10.4) for household contacts, compared to other contacts; adjusted OR 0.32 (0.14–0.74) for HIV-positive contacts compared to HIV-negative contacts. These results were not altered by adjusting for degree of smear positivity of PSCs or for the other factors shown in Table 2. The results were similar if all index cases with <5 bands were excluded.

**Table 2:** Probability of transmission from a smear-positive putative source contact (PSC) being confirmed by restriction fragment length polymorphism, according to characteristics of case and PSC

Characteristic		Confirmed/ total pairs (%)	p	Odds ratio, 95% confidence interval (adjusted for closeness of contact)
Closeness of contact				
	Same household	22/50 (44.0)		3.6 (1.4–9.3)
	Close family, not household	17/38 (44.7)		3.5 (1.5–8.6)
	Other	10/55 (18.2)	0.006	Referent
Time between diagnosis of disease in PSC and case-patient (mo)				
	<12	21/62 (33.9)		Referent
	12–23	13/36 (36.1)		1.1 (0.44–2.6)
	>24	15/45 (33.3)	1.0	0.88 (0.37–2.1)
Age of case-patient (y)				
	<30	20/53 (37.7)		Referent
	30–44	23/67 (34.3)		0.72 (0.32–1.6)
	>45	6/23 (26.1)	0.3 (trend)	0.48 (0.17–1.3)
Age of PSC (y)				
	<30	20/49 (40.8)		Referent
	30–44	21/63 (33.3)		0.93 (0.42–2.1)
	>45	8/31 (25.8)	0.2 (trend)	0.59 (0.19–1.8)
Sex of case-patient				
	Female	28/81 (34.6)	0.9	Referent
	Male	21/62 (33.9)		1.2 (0.56–2.4)
Sex of PSC				
	Female	33/75 (44.0)	0.01	Referent
	Male	16/68 (23.5)		0.46 (0.21–0.99)
HIV status of case-patient				
	HIV negative	16/37 (43.2)	0.1	Referent
	HIV positive	19/67 (28.4)		0.55 (0.23–1.3)
HIV status of PSC				
	HIV negative	27/59 (45.8)	0.01	Referent
	HIV positive	15/62 (24.2)		0.32 (0.14–0.74)
Drug resistance of PSC				
	None	42/129 (32.6)	0.2	Referent
	Isoniazid resistant	7/14 (50.0)		2.1 (0.66–6.8)
Smear positivity of PSC				
	1+	5/18 (27.8)		Referent
	2+	12/36 (33.3)		0.94 (0.26–3.5)
	3+	12/43 (27.9)		0.89 (0.25–3.2)
	4+	20/46 (43.5)	0.4	1.6 (0.46–5.4)

To estimate the origin of the infection in those for whom transmission from identified, PSCs was not confirmed, cases were classified as likely to be due to reactivation if the strain was the first or only example in the dataset and as recent infection if the strain was part of an existing cluster. For the patients without confirmed transmission from their PSCs, 33% had first/unique strains. In the whole dataset, the proportion of persons with first/unique strains was 39%, or 33% after excluding the first 2 years, in which first examples are more likely.

### Proportion of TB Cases Due to Recognized Close Contact with a Smear-positive Patient

Of the 1,029 TB patients included in the study, 219 (21.3%) had at least 1 named family or household PSC with recorded smear-positive tuberculosis, and 86 other patients reported a PSC who was not identified in the database who may have had smear-positive disease. Overall, 177 (17.2%) of the patients had at least 1 PSC outside the family or household. Other patients either had no PSCs or none with smear-positive disease. Taking the proportion with transmission confirmed from family and household PSCs combined as 44.3% (Table 2) and the prevalence of exposure (at least 1 family or household PSC with smear-positive TB) as 21.3%, we estimate that 0.443 × 0.213 = 9% of TB case-patients in this population were attributable to recent transmission from identified smear-positive PSCs in their families or households. If the 86 additional PSCs are included, the estimate rises to 13%. Similarly, we estimate a PAF of 3.1% (0.182 × 0.172) for recent transmission from identified PSCs outside the family and household.

### Proportion of TB Cases Due to Transmission from HIV-positive Patients

HIV-negative PSCs were twice as likely as HIV-positive PSCs to be confirmed by RFLP as sources of infection (46% vs. 24%), and this was seen both within the family and household (64% vs. 32%) and outside (23% vs. 12%). Overall, 61% of smear-positive TB patients were HIV positive. If we assume that the pattern of transmission from contacts is representative of the relative transmission from HIV-positive and HIV-negative patients in other settings, 45% of *M. tuberculosis* infections in this community are transmitted from HIV-positive patients: (0.61 × 0.24)/[(0.61 × 0.24) + ([1–0.61] × 0.46)].

### Investigation of Clusters

Cluster sizes ranged from 2 to 37. The proportion of patients with clustered strains for whom epidemiologic links were identified is shown in Table 3. This proportion was no higher for strains with high band numbers than for those with <5 bands and did not vary consistently with cluster size. If we assume 1 index case per cluster, 623 of the 754 clustered cases were secondary. Of the 84 cases with epidemiologic links within RFLP-defined clusters, 52 were secondary, so sources of infection were identified for 8.3% (52/623) of secondary cases within clusters.

**Table 3:** Proportion of clustered strains with epidemiologic links

		No. with epidemiologic links within a cluster	Total with clustered strains	% with link
Total		84	754	11.1
No. bands				
	>5	72	679	10.6
	<5	12	75	16.0
Cluster size (strains with >5 bands only)				
	Cluster 2–4	18	187	9.6
	Cluster 5–9	24	152	15.8
	Cluster >10	30	340	8.8

## Conclusion

In Africa, case finding for TB is generally passive. Although being a household contact of a TB patient is a strong risk factor, in Africa as elsewhere (*15**,**20*), in high-incidence settings, most cases of TB are not attributable to household contact. This finding has been demonstrated in traditional epidemiologic studies (*21*) and more recently by using molecular techniques (*22*). The apparent importance of casual contact in TB transmission is not surprising since many people exposed to a small risk can account for more disease than a few exposed to a large risk (*23*).

DNA fingerprinting allows direct measurement of the proportion of cases with known exposure who acquired TB from that exposure. This proportion has varied from 95% in the Netherlands (*10*), 70% in San Francisco (*8*) and 71% elsewhere in the United States (*9*), to <50% in Cape Town, South Africa (*24*), and in our study. The studies varied in whether they included smear-negative PSCs, in the way contact was defined, and in whether similar but not identical RFLP patterns were included. Smear-negative PSCs were associated with a lower likelihood of confirmed transmission in our study and in the United States (*9*). Workplace contacts in the United States (*9*) and contacts outside the family and household in our study were much less likely to be confirmed as sources of infection. The inclusion of similar RFLP patterns that are the first example of their type will increase the proportion of confirmed transmissions, though with an increased risk of false attributions of the source of infection. Even identical strains may have other origins, particularly if the strain is common. On the other hand, actual transmission may not be recognized if different strains are seen in the PSC and case because of cross-contamination or other laboratory error or because the infection in the PSC was a mixture of strains.

This analysis, like all analyses to date of *M. tuberculosis* transmission based upon IS6*110* RFLP patterns, is based on the assumption that multiple infections are infrequent and thus that a single RFLP-defined strain reflects the infection status of a patient. A recent study from South Africa has hinted that multiple infections may be more frequent than previously assumed (*25*). If this is the case, then we (and all previous RFLP-based studies) have underestimated the proportions of transmission occurring within households. This assumption should not affect our estimates of relative transmission from HIV-positive and HIV-negative patients.

A lower proportion of confirmed transmission from identifiable PSCs is expected in high-incidence settings. As the incidence of TB decreases in a population, patients are increasingly concentrated in high-risk groups with particular risk factors for disease. Close contacts of patients may share many risk factors. As the risk of a close contact of a TB patient having had TB increases relative to the risk of a casual contact having had TB, the proportion of TB due to transmission from a close contact will also increase. In our study, 44% of patients with a smear-positive PSC in their household within the previous few years appear to have acquired their infection from that person. The source of the infections in persons without confirmed transmission is unknown, but two thirds of patients were part of existing clusters so the infections probably were recently acquired locally.

In Cape Town, the proportion of identifiable household contacts with confirmed transmission was similarly low (*24*). In that study, the proportion of TB in the community attributable to transmission within the household (from smear-positive or smear-negative contacts) was estimated at 19%. In our study, by using different methods, we estimated that 9%–13% of TB cases were attributable to transmission from smear-positive PSCs within the household or close family, and 3% from other named sources. The low proportion of cases with identified sources of infection is corroborated by conventional epidemiologic studies in this and other populations (*15**,**21*).

The low proportion of TB attributable to identifiable links is also supported by the similarly low proportion of persons in clusters who can be linked epidemiologically, both in our study and studies in South Africa (*22*) and India (*26*). In our study, the epidemiologic links were established independently of the molecular data; further links might have been found by detailed investigations of particular clusters (*10*), but many of these more-difficult-to-define links represent casual contact. Identifiable links have been found for a higher proportion of clustered patients in low-incidence settings (*10**,**27**,**28*), but, excluding the casual links, the proportion is still <50%.

One reason for the particularly low proportion of confirmed transmission in our study is the high prevalence of HIV and the effect of HIV on transmission. Our study is the first to demonstrate lower infectiousness of HIV-infected TB patients by DNA fingerprinting of epidemiologically linked case-contact pairs. The lower rate of transmission persisted after adjusting for degree of smear positivity. Although HIV status was not available for all patients, this factor should not bias this estimate.

Extrapolating the results from case-PSC pairs to the community assumes similar relative transmission patterns, but is reasonable since HIV-positive patients had similarly reduced transmission within families and households and outside them. The lower infectiousness of HIV-positive patients does not mean that they have a minor role in TB transmission, since nearly two thirds of TB patients are HIV positive. It does, however, help limit the extent of the HIV-related increase in TB in the population (*1**,**11**,**29*).
